# Adjuvant Chemotherapy Is Associated with Improved Survival in Advanced Ampullary Adenocarcinoma—A Population-Based Analysis by the German Cancer Registry Group

**DOI:** 10.3390/jcm14113869

**Published:** 2025-05-30

**Authors:** Jannis Duhn, Julia Strässer, Lennart von Fritsch, Rüdiger Braun, Kim C. Honselmann, Markus Kist, Thaer S. A. Abdalla, Kees Kleihues-van Tol, Bianca Franke, Fabian Reinwald, Andrea Sackmann, Bernd Holleczek, Anna Krauß, Monika Klinkhammer-Schalke, Sylke R. Zeissig, Steffen Deichmann, Tobias Keck, Ulrich F. Wellner, Louisa Bolm

**Affiliations:** 1 Department of Surgery, University Medical Center Schleswig-Holstein, Campus Lübeck, Ratzeburger Allee 160, 23562 Lübeck, Germanyruediger.braun@uksh.de (R.B.); steffen.deichmann@uksh.de (S.D.);; 2 Network for Care, Quality and Research in Oncology, German Cancer Registry Group of the Association of German Tumor Centers (ADT), 14057 Berlin, Germanymonika.klinkhammer-schalke@klinik.uni-regensburg.de (M.K.-S.); sylke.zeissig@lgl.bayern.de (S.R.Z.); 3 Cancer Registry of Rhineland-Palatinate in the Institute for Digital Health Data, 55101 Mainz, Germany; 4 Hessian Cancer Registry, Hessian Office for Health and Care, 60439 Frankfurt am Main, Germany; 5 Cancer Registry Saarland, 66117 Saarbrücken, Germany; 6 Cancer Registry Mecklenburg-Western Pomerania, c/o Institute for Community Medicine, University Medicine Greifswald, 17489 Greifswald, Germany; 7 Institute of Clinical Epidemiology and Biometry (ICE-B), University of Würzburg, 97070 Würzburg, Germany

**Keywords:** ampullary adenocarcinoma (AMPAC), adjuvant chemotherapy, real-world data

## Abstract

**Introduction**: Ampullary adenocarcinomas (AMPACs) represent rare malignant neoplasms arising in the Ampulla of Vater. Due to a lack of prospective studies and heterogeneous results from retrospective analyses, the outcomes of adjuvant chemotherapy (AC) in AMPAC are unclear. **Methods**: Pooled, pseudonymized data were retrieved from clinical cancer registries participating in the German Cancer Registry Group of the Association of German Tumor Centers (GCRG/ADT). Patients who underwent surgical resection of AMPACs (ICD-10: C24.1) with subsequent follow-up or AC were included. Patients with 90-day postoperative mortality were excluded. The epidemiologic and histopathologic features as well as the overall survival and recurrences were compared in both groups using R statistics. **Results**: In total, 830 patients with AMPACs were identified, of which 184 (22.2%) received AC. The surgery + AC patients showed more advanced tumor stages and more pronounced locoregional invasion as compared to the group undergoing surgery alone. AC was independently associated with an improved overall survival (OS) in a multivariable analysis (HR 0.57, *p* < 0.001), where pT3-4 status, lymph node metastases, vascular invasion, and advanced grading remained independent prognostic factors for OS. In the subgroup analyses, AC was associated with improved OS in the patients with pT3-4 tumors, lymph node metastases, lymphovascular invasion, and advanced grading, or UICC stage III, whereas no association with the OS was observed in the other subgroups. AC was also associated with superior disease-free survival (DFS) in a multivariable analysis. **Conclusions**: We provide a large-scale population-based analysis of AMPAC patients, showing an association of AC with improved OS in patients with advanced-staged disease or signs of locoregional invasion as compared to surgery alone.

## 1. Introduction

Periampullary cancers are rare malignant neoplasms that arise in the complex anatomical region around the Ampulla of Vater. These cancers can be anatomically classified into pancreatic ductal adenocarcinoma (60%), distal bile duct carcinoma (10%), duodenal adenocarcinoma (10%), and ampullary adenocarcinoma (AMPAC; 20%) [1].

AMPACs originate from the epithelial junction in the major duodenal papilla [2]. Despite being associated with improved survival rates as compared to other periampullary cancers, the long-term survival remains poor. The reported median overall survival (OS) reaches up to 64 months, and median disease-free survival (DFS) can reach up to 85 months [3,4,5]. Complete oncological resection remains the only potentially curative option and should be performed if feasible [6].

After curative intent resection, the US-American National Comprehensive Cancer Network (NCCN) guidelines state that all patients can receive n adjuvant therapy, mainly by means of adjuvant chemotherapy (AC) with or without chemoradiation or, alternatively, undergo surveillance in stage I and II disease [6]. This reflects heterogeneous results from mainly smaller retrospective analyses. An analysis from the National Cancer Database (NCDB) shows a superior overall survival (OS) associated with AC [7], whereas other studies failed to detect an association of AC with OS [8,9]. Several other studies have shown improved OS following AC in patients with advanced-stage disease or negative prognostic factors only [10,11].

Regarding this controversial evidence, there is a clinical need to better define outcomes following AC in AMPAC patients in large-scale real-world patient cohorts in order to better tailor adjuvant therapy.

In the present analysis, we assess the outcomes following AC in AMPAC patients using population-based data derived from regional clinical cancer registries participating in the German Cancer Registry Group of the Association of German Tumor Centers (GCRG/ADT). The aim of the present study is to understand the role of AC for long-term outcomes in a large real-world German AMPAC cohort.

## 2. Methods

### 2.1. Study Population

This observational study was approved by the ethics committee of the University of Luebeck, Germany (ethical approval number #2023-156; 17 August 2020), and performed in accordance with the data use regulations of the GCRG/ADT. This retrospective, population-based study was conducted using data retrieved from regional clinical cancer registries and other cancer centers. The GCRG/ADT regularly collects nationwide data from regional clinical cancer registries and establishes a pooled pseudonymized dataset for analysis. This dataset was queried for patients being diagnosed with AMPAC (ICD-10: C24.1) receiving upfront curative intent pancreatoduodenectomy or total pancreatectomy with or without adjuvant chemotherapy. Patients receiving adjuvant therapy other than chemotherapy and patients with neoadjuvant therapy were excluded. In addition, we excluded patients with 90-day postoperative mortality, to avoid an immortal time bias in patients receiving AC (Supplementary Figure S1).

### 2.2. Study Parameters

The following variables were extracted for analysis: Age at diagnosis, sex, pathological workup including tumor size (pT) and lymph nodes (pN), lymph (L) and blood vessel (V) invasion, tumor grading according to Broders, as well as resection margins (R). The follow-up included time to follow-up (in months) from diagnosis and the status at last follow-up (alive/dead for overall survival (OS), or disease-free/progressed for disease-free survival (DFS), respectively).

### 2.3. Handling of Missing Data

As is common in retrospective datasets, certain co-variables had missing values in up to 30% of the cases. Overall, at least one missing value was found in the variables of interest in 59% of all selected cases. To account for this, we performed multiple imputations by chained equations (MICE) [12], using 59 imputations with 10 iterations, utilizing the “mice” package (version 3.17.0) in R (R Foundation for Statistical Computing, Vienna, Austria, version 4.4.2) with R Studio (Posit Software, PBC, Boston, MA, USA, version 2024.09.1+394). After multiple imputations, the results were pooled into one dataset for analysis.

### 2.4. Statistical Analysis

Whole data processing and statistical analysis was performed in R with R Studio. A Kolmogorov–Smirnov test was conducted, showing a non-normal distribution of “Age”, representing the only continuous variable reported in this study. For descriptive statistics of continuous and categorical variables, the median with interquartile range (IQR) and absolute numbers with percentage of total were calculated, respectively. Non-parametric testing with the Mann–Whitney U test (equivalent to Kruskal–Wallis or Wilcoxon rank sum) was conducted to assess differences in age. Dependencies of categorical variables were analyzed using Pearson’s chi-squared test.

To assess OS and DFS, the Kaplan–Meier method and log-rank test were used in the univariable analysis. To account for covariable imbalance in retrospective datasets, multivariable Cox proportional hazards models were fitted to assess the prognostic impact of independent variables. A multivariable binary logistic regression was performed to evaluate predictive factors for disease recurrence. The significance level throughout this study was set to *p* < 0.05 (two-sided) and confidence intervals (CIs) are reported as 95% CI.

## 3. Results

### 3.1. Receival of AC Is Associated with Advanced-Stage Disease

Using the inclusion criteria, we identified 830 patients diagnosed with AMPAC from 2000 to 2023 receiving upfront oncologic pancreatic resection. The median age at diagnosis was 70 (61–77) years, and most patients were of male sex (59.9%). R0 resections were achieved in almost all of the patients (97.7%). After oncologic resection, 184 patients (22.2%) were treated with AC (Supplementary Table S1).

Epidemiological and histopathological features were compared between patients receiving surgery + AC and surgery alone (Table 1). Patients receiving AC were younger (65 vs. 72 years, *p* < 0.001), but sex was comparable, with male predominance in both groups. Furthermore, the patients receiving AC had more advanced tumor stages, with larger tumor sizes (pT3-4: 68.5 vs. 47.7%, *p* < 0.001), and more frequent node-positive disease (pN+: 66.3 vs. 41.5%) in comparison to the surgery alone group. Moreover, the patients receiving AC had more undifferentiated tumors (G3-4: 42.4 vs. 25.5%, *p* < 0.001), as well as lymphovascular (62.5 vs. 40.2%, *p* < 0.001) and vascular invasion (16.8 vs. 7.7%, *p* < 0.001). In contrast, the R0 resection rates were comparable in both groups (96.2 vs. 98.1%, *p* = 0.242).

The chemotherapy regimens used in AC were diverse, with a predominance of Gemcitabine (38.6%)- and Capecitabine (16.3%)-based therapies. FOLFIRINOX was used in 14.1% of the patients. In 38 patients (20.7%), the chemotherapy regimen was not reported (Supplementary Table S2).

### 3.2. AC Is Associated with Improved OS in Multivariable Analysis

**Figure 1:**
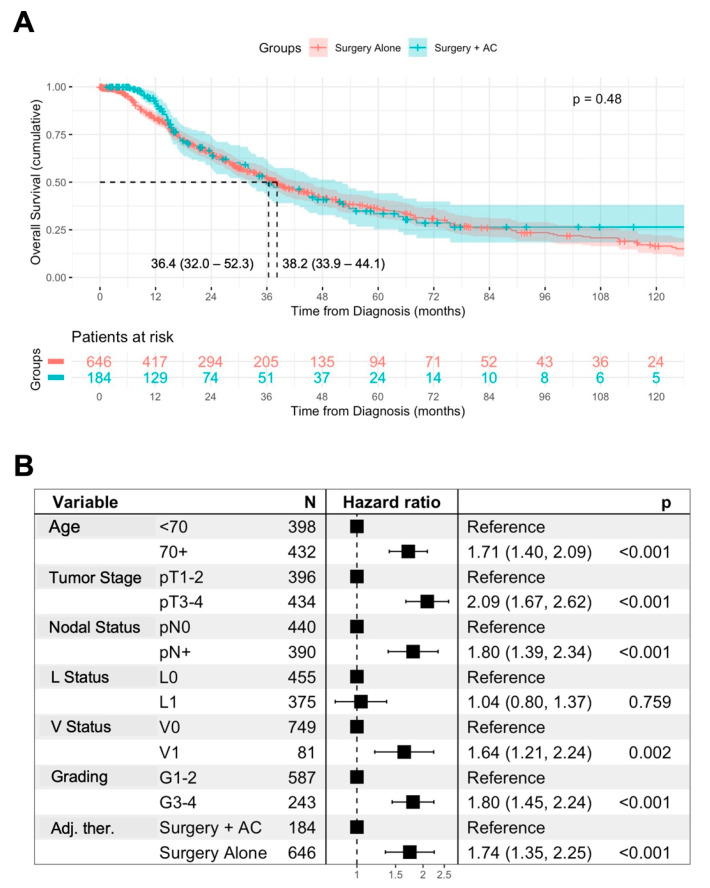
**Association of AC with overall survival.** Univariable analysis of the overall survival (OS) from diagnosis in patients treated with surgery alone or surgery + AC, using Kaplan–Meier method with log-rank test. Displayed is the median OS with 95% confidence interval (**A**). Multivariable Cox regression analysis of prognostic factors for OS including AC in AMPAC patients (**B**).

In univariable analysis, surgery + AC was not associated with improved OS in comparison to surgery alone (median OS: 36.4 vs. 38.2 months, *p* = 0.48) (Figure 1A). However, regarding more advanced tumor stages, this might result from a retrospective selection bias. We therefore assessed prognostic factors for OS in AMPAC patients. An age ≥ 70 years, pT3-4 staged tumors, lymph node metastases, and lymphovascular and vascular invasion as well as higher grading were associated with impaired OS in the patients (Supplementary Figure S2). In contrast, the resection margins were not associated with OS, potentially due to the low sample sizes in the R+ group (Supplementary Figure S2H).

To further assess the impact of AC on the OS of the patients, we conducted a multivariable Cox regression analysis including previously identified prognostic factors (Figure 1B). In this analysis, an age ≥ 70 years (HR 1.71, *p* < 0.001), pT3-4 status (HR 2.09, *p* < 0.001), the presence of lymph node metastases (HR 1.8, *p* < 0.001), vascular invasion (HR 1.64, *p* = 0.002), and advanced grading (HR 1.8, *p* < 0.001) were retained as independent negative prognostic factors for the OS of the patients. Furthermore, surgery + AC was independently associated with improved OS in comparison to surgery alone (HR 0.57, *p* < 0.001).

### 3.3. AC Is Associated with Improved OS in Patients with Negative Prognostic Factors

**Figure 2:**
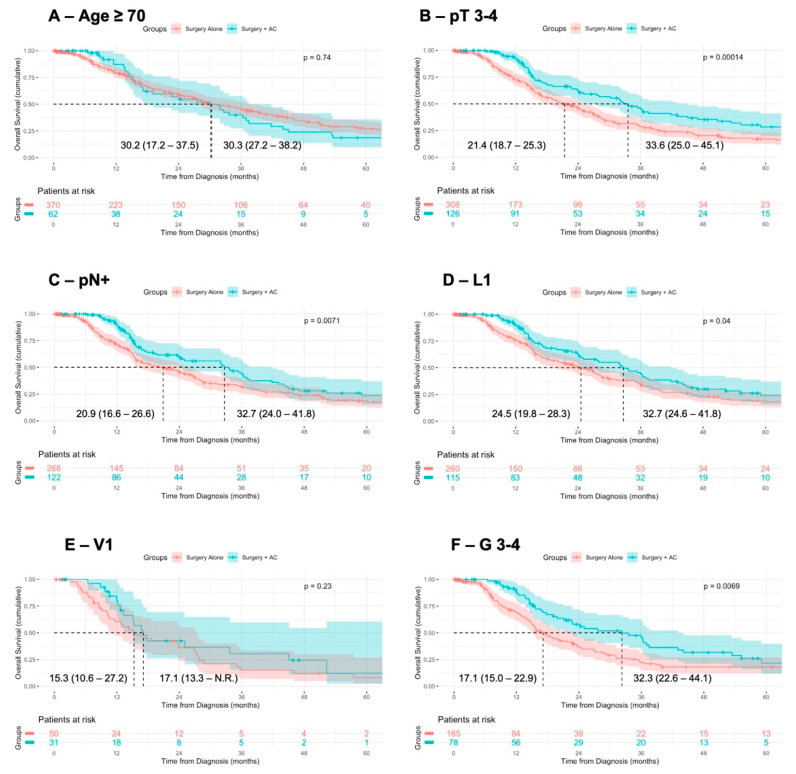
**Association of AC with OS in patients with negative prognostic factors.** Comparison of overall survival from diagnosis in patients with negative prognostic factors receiving surgery alone or surgery + adjuvant therapy, using Kaplan–Meier method with log-rank test. Displayed is the median overall survival with 95% confidence interval.

Having observed an association of AC with improved OS after adjustment for confounding variables, we stratified the patients according to previously identified negative prognostic factors. In the univariable analysis, compared to surgery alone, surgery + AC was associated with improved OS in the patients with pT3-4 staged tumors (median OS: 33.6 vs. 21.4 months, *p* < 0.001), lymph node metastases (median OS: 32.7 vs. 20.9 months, *p* = 0.007), lymphovascular invasion (32.7 vs. 24.5 months, *p* = 0.04), and undifferentiated tumors (median OS 32.3 vs. 17.1 months, *p* = 0.007) (Figure 2). Vice versa, in the patients stratified according to positive prognostic factors, surgery + AC was not associated with improved OS in comparison to surgery alone (Supplementary Figure S3).

**Figure 3:**
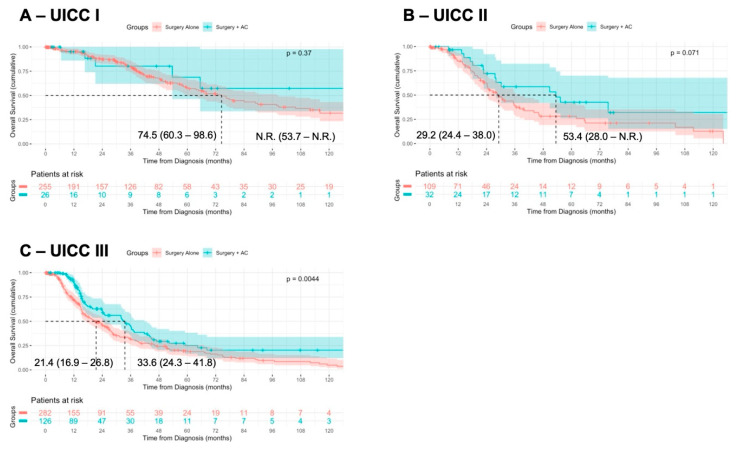
**Association of surgery + AC in UICC stage-matched cohorts**. Patients were stratified according to UICC stage I (pT 1–2, pN0; (**A**)), UICC stage II (pT3, pN0; (**B**)), and UICC stage III (pT4 and/or pN+; (**C**)) and the OS was compared in patients receiving surgery alone or surgery + AC, using Kaplan–Meier method with log-rank test.

We further stratified the patients according to UICC stage I (pT 1–2, pN0), stage II (pT3, pN0), and stage III (pT4 and/or pN+) and assessed OS following surgery + AC or surgery alone. In UICC stage I (median OS not reached vs. 74.5 months, *p* = 0.37) and UICC stage II (median OS: 53.4 vs. 29.2 months, *p* = 0.07), the OS was not significantly different, although a tendency towards a superior OS following surgery + AC was observed in UICC stage II (Figure 3A,B). However, in UICC stage III, surgery + AC was associated with a prolonged OS of the patients (median OS: 33.6 vs. 21.4 months, *p* = 0.004) (Figure 3C).

### 3.4. Locoregional Invasion Is Associated with Disease Recurrence

Disease recurrence by means of distant or locoregional recurrence was documented in 170 patients (20.5%). The patients experiencing recurrence were significantly younger (mean age 68 vs. 70 years, *p* = 0.045) and showed signs of advanced locoregional tumor invasion, by means of larger tumors, more frequent lymph node metastases, and higher grading, as well as more frequent lymphovascular and vascular invasion. In contrast, positive resection margins were not significantly associated with disease recurrence, although the sample sizes were low (Table 2). The patients experiencing disease recurrence more often received surgery + AC in comparison to the patients without recurrence (35.9 vs. 18.6%, *p* < 0.001).

### 3.5. AC Is Independently Associated with Disease-Free Survival

**Figure 4:**
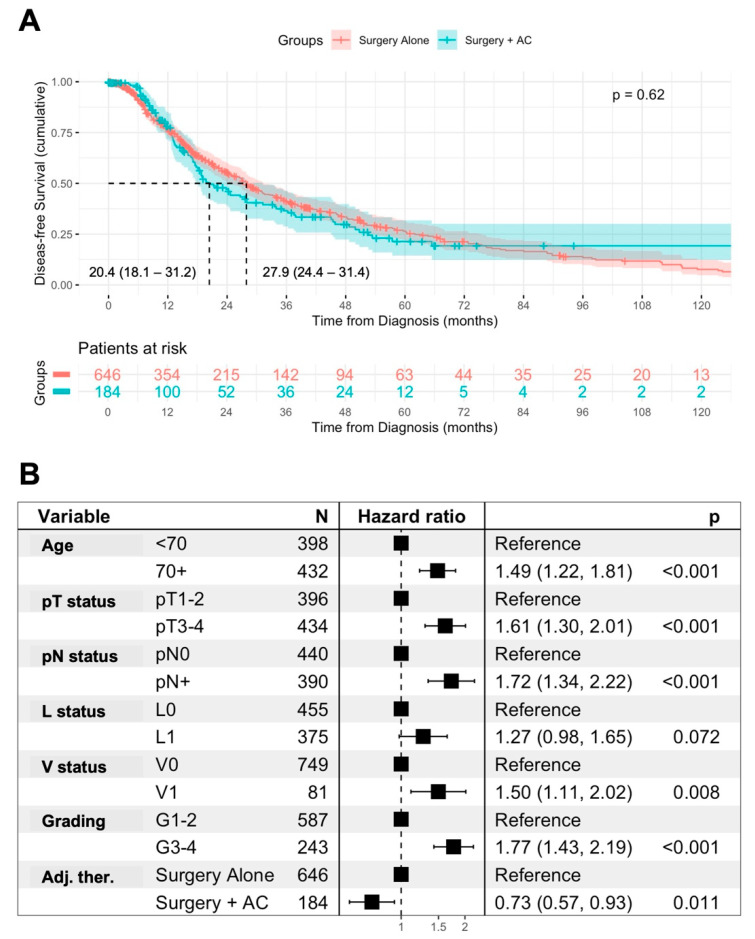
**Association of AC with disease-free survival.** Univariable analysis of the disease-free survival (DFS) from diagnosis in patients treated with surgery alone or surgery + AC, using Kaplan–Meier method with log-rank test. Displayed is the median DFS with 95% confidence interval (**A**). Multivariable Cox regression analysis of prognostic factors for DFS including AC in AMPAC patients (**B**).

In the univariable analysis, surgery + AC was not associated with disease-free survival (DFS) in comparison to surgery alone (median DFS: 20.4 vs. 27.9 months, *p* = 0.62) (Figure 4A). However, in a multivariable Cox regression analysis including age and prognostic histopathological features, surgery + AC was independently associated with DFS in comparison to surgery alone (HR 0.73, *p* = 0.011). An age ≥ 70 years (HR 1.49, *p* < 0.001), pT3-4 staged tumors (HR 1.61, *p* < 0.001), lymph node metastases (HR 1.72, *p* < 0.001), vascular invasion (HR 1.5, *p* = 0.008), and advanced grading (HR 1.77, *p* < 0.001) remained independent prognostic factors for DFS in patients (Figure 4B).

In patients stratified according to negative prognostic factors for DFS, receiving AC was only associated with improved DFS in comparison to surgery alone in the patients with pT3–4 tumors (median DFS 18.8 vs. 16.8 months, *p* = 0.02) and advanced grading (median DFS: 19.0 vs. 13.9 months, *p* = 0.032) (Supplementary Figure S4).

## 4. Discussion

AMPACs represent the second most common cancer in the periampullary region and are found in up to 20% of all pancreatic head resection specimens for suspected malignancy [13]. Due to the junction of different epithelia in the Ampulla of Vater, AMPACs can be further subclassified into intestinal (INT), pancreatobiliary (PB), and mixed histology [14]. A meta-analysis conducted by Shin et al. revealed a shorter OS in PB-AMPAC over INT-AMPAC, also presenting in more advanced tumor stages with pronounced signs of locoregional tumor invasion [15]. Despite the PB subtype, signs of locoregional invasion, e.g., pT3-4 status, lymph node metastases, lymphovascular invasion, and perineural invasion, were identified as relevant prognostic factors in AMPACs [15,16].

The current treatment guidelines issued by the US-American NCCN recommend surgical resection if feasible; however, no clear recommendation on which patients should receive AC is issued due to the lack of prospectively designed studies [6]. Most retrospective studies showed an association of AC with superior OS in patients with negative prognostic factors, but not in the unstratified AMPAC cohort [8,9,10,11,16,17].

In the present study, we identified patients receiving upfront surgical resection for AMPACs, using population-based data derived from regional clinical cancer registries. We included 830 patients, of which 184 (22.2%) received AC for further analysis. The AC administration rates were lower as compared to other retrospective analyses ranging from 30 to 60% [9,10], potentially reflecting a lack of clear recommendations [6]. However, an underreporting of AC treatment to the cancer registries cannot be ruled out.

We observed more advanced tumor stages in the patients receiving AC, especially pronounced signs of locoregional tumor invasion, e.g., lymphovascular invasion and higher grading, indicating the preferred selection of patients with advanced tumors for AC while favoring surveillance in the early tumor stages. This patient selection strategy was also observed in an analysis from the US-American NCDB [9].

We were able to confirm pronounced tumor stages and locoregional invasion as relevant prognostic factors for the OS of AMPAC patients. In addition, elevated CA 19-9 levels were previously identified as prognostic factors [16] alongside the PB subtype [15]. Unfortunately, neither the CA 19-9 levels nor histological subtyping of AMPACs were present in the dataset for analysis, serving as a critical limitation to our study.

In a univariable analysis, AC was not associated with the OS of patients. However, this might result from a retrospective selection bias, reflecting more advanced tumors in the surgery + AC cohort. We therefore conducted a multivariable Cox regression including previously identified prognostic factors. After adjusting for confounding factors, AC was independently associated with the improved OS of patients (HR 0.75, *p* < 0.001).

To further evaluate the effect of AC, the patients were stratified according to the presence of previously identified negative prognostic factors. In these analyses, AC was associated with an improved OS in patients presenting with pT3-4 staged tumors, the presence of lymph node metastases, lymphovascular invasion, and advanced tumor grading (G3-4). Vice versa, AC was not associated with improved OS in patients with positive prognostic factors. These results are in line with previous retrospective studies including those from our group, showing a survival benefit following AC in patients with negative prognostic factors, while failing to show a survival benefit in the early stages or the INT subtype [10,17,18].

The NCCN guidelines state that AC or observation can be performed in UICC stage I and II, whereas AC with or without radiation can be performed in stage III, not mentioning the option of observation in these patients [6]. Therefore, we stratified the patients according to their UICC stages. We observed an association of AC with an improved OS only in UICC stage III, thus supporting the recommendation issued by the NCCN [6].

Few studies have evaluated prognostic factors for recurrence in AMPAC patients. A retrospective multicenter cohort study identified only lymph node metastases as an independent prognostic factor for DFS in patients [19], whereas other studies also identified the PB subtype [20] or advanced T stage and a higher grading as prognostic factors [21]. Likewise, in our analysis, advanced tumor stages and signs of locoregional invasion were associated with disease recurrence. In addition, patients with disease recurrence received AC more often, potentially due to the previously discussed patient selection. However, AC was not associated with inferior DFS compared to surgery alone in a univariable analysis. In a multivariable analysis, AC was independently associated with the superior DFS of the patients after adjustment for confounding variables, on the contrary to previous studies [19]. Namely, an age ≥ 70 years, pT3-4 status, lymph node metastases, hemangioinvasion, and an advanced grading remained independent negative prognostic factors for DFS. Patterns of recurrence and risk factors for certain types of recurrence in AMPACs are as of yet insufficiently understood and warrant further investigation, while being out of scope of this current project. In the patients with larger tumors and advanced gradings, the receival of AC was associated with improved DFS. Therefore, AC seems sufficient for the prevention of recurrence and prolonging DFS and OS in selected AMPAC patients with signs of locoregional invasion and tumor dedifferentiation.

This study has several limitations. Due to its retrospective design, a selection bias for patients receiving surgery alone or surgery + AC cannot be fully excluded, despite accounting for this issue using multivariable analyses and stage-matched cohorts for the subgroup analyses. In addition, the design of this registry-based study does not allow for source validation of data. However, it is accepted that clinical registry data give valuable insight into real-world epidemiology, treatment, and outcomes of patients.

Importantly, missing information on relevant prognostic factors, e.g., the histological subtype or CA 19-9 levels, further limits the evaluation of prognostic factors and might interfere with regression analyses. Furthermore, missing records on the PB versus INT subtype in the present cancer registry data may represent a confounder in our analysis. However, we provided granular information on histopathological features and subgroup analyses to account for this issue. Likewise, granular information on the performance status of patients was not available in most patients and was therefore not considered in our study, which might have influenced the clinical decision to perform surgery + AC. In addition, incomplete data on chemotherapy regimes in 21% of the cases and missing data on the dosages and number of cycles received, as well as the high heterogeneity in chemotherapy regimens, limits the possibility to directly compare different perioperative regimes.

## 5. Conclusions

As to our knowledge, we provide the first population-based analysis on prognostic factors for OS and DFS and the outcomes of AC in a large cohort of resected AMPAC patients. The present study disclosed an association of AC with prolonged survival in patients with advanced tumors and signs of locoregional invasion, e.g., lymph node metastases, lymphovascular invasion, undifferentiated tumor grading, or UICC stage III. In other subgroups, AC was not associated with prolonged survival, potentially reinforcing a patient selection bias for AC versus surgery alone. These results further emphasize the need for patient selection when evaluating AC in AMPACs.

## Figures and Tables

**Table 1:** **Factors associated with AC.** Statistical significance tested using chi-squared test (categorical variables) or Mann–Whitney U test (continuous variables).

Factors Associated with AC	Surgery Alone	Surgery + AC	
	n = 646	n = 184	
**Parameter**	n (%)/ median (IQR)	n (%)/ median (IQR)	*p*-value
**Age at diagnosis (years)**	72 (63 to 78)	65 (58 to 72)	** <0.001 **
**Sex**			0.692
female	262 (40.6)	71 (38.6)	
male	384 (59.4)	113 (61.4)	
**T stage**			**<0.001**
T1	123 (19.0)	8 (4.3)	
T2	214 (33.1)	50 (27.2)	
T3	259 (40.1)	105 (57.1)	
T4	49 (7.6)	21 (11.4)	
**N stage**			**<0.001**
N0	378 (58.5)	62 (33.7)	
N1	203 (31.4)	88 (47.8)	
N2	65 (10.1)	34 (18.5)	
**UICC stage**			**<0.001**
UICC I	255 (39.5)	26 (14.1)	
UICC II	109 (16.9)	32 (17.4)	
UICC III	282 (43.7)	126 (68.5)	
**Grading**			** <0.001 **
G1–2	481 (74.5)	106 (57.6)	
G3–4	165 (25.5)	78 (42.4)	
**Lymphovascular invasion**			** <0.001 **
L0	386 (59.8)	69 (37.5)	
L1	260 (40.2)	115 (62.5)	
**Vascular invasion**			** <0.001 **
V0	596 (92.3)	153 (83.2)	
V1	50 (7.7)	31 (16.8)	
**R status**			0.242
R0	634 (98.1)	177 (96.2)	
R1	9 (1.4)	6 (3.3)	
R2	3 (0.5)	1 (0.5)	

**Table 2:** **Factors associated with recurrence in AMPAC patients.** Statistical significance tested using chi-squared test (categorical variables) or Mann–Whitney U test (continuous variables).

Factors Associated with Recurrence	No Recurrence	Recurrence	
	n = 660	n = 170	
**Parameter**	n (%)/ median (IQR)	n (%)/ median (IQR)	*p*-value
**Age (years)**	70 (62 to 77)	68 (60 to 76)	**0.045**
**Gender**			0.635
female	268 (40.6)	65 (38.2)	
male	392 (59.4)	105 (61.8)	
**T stage**			**<0.001**
T1	118 (17.9)	13 (7.6)	
T2	220 (33.3)	44 (25.9)	
T3	283 (42.9)	81 (47.6)	
T4	38 (5.8)	32 (18.8)	
**N stage**			**<0.001**
N0	389 (58.9)	51 (30.0)	
N1	203 (30.8)	88 (51.8)	
N2	68 (10.3)	31 (18.2)	
**UICC stage**			**<0.001**
UICC I	256 (38.8)	25 (14.7)	
UICC II	119 (18.0)	22 (12.9)	
UICC III	285 (43.2)	123 (72.4)	
**Grading**			**<0.001**
G1–2	488 (73.9)	99 (58.2)	
G3–4	172 (26.1)	71 (41.8)	
**Lymphovascular invasion**			**<0.001**
L0	398 (60.3)	57 (33.5)	
L1	262 (39.7)	113 (66.5)	
**Vascular invasion**			**0.004**
V0	606 (91.8)	143 (84.1)	
V1	54 (8.2)	27 (15.9)	
**R status**			0.155
R0	648 (98.2)	163 (95.9)	
R1	10 (1.5)	5 (2.9)	
R2	2 (0.3)	2 (1.2)	
**Adj. Chemotherapy (AC)**			**<0.001**
surgery alone	537 (81.4)	109 (64.1)	
surgery + AC	123 (18.6)	61 (35.9)	

## Data Availability

The data were obtained from the German Cancer Registry Group of the Association of German Tumor Centers and are available upon request from and under regulations of the Association of German Tumor Centers and specific state regulations.

## Supplementary Materials

The following supporting information can be downloaded at https://www.mdpi.com/article/10.3390/jcm14113869/s1.
