# Supplementary material for: Adjuvant Chemotherapy Is Associated with Improved Survival in Advanced Ampullary Adenocarcinoma—A Population-Based Analysis by the German Cancer Registry Group
Source: J Clin Med. 2025 May 30;14(11):3869. doi: 10.3390/jcm14113869 (PMC12155577; doi:10.3390/jcm14113869)
Supplement: Supplementary file 1 [file jcm-14-03869-s001.zip › jcm-3628915-supplementary.pdf]

## Supplementary information

### Supplementary tables

**Supplementary Table S1: Epidemiological and histopathological characteristics of the study cohort.**

| AMPAC | n = 830 |
|---|---|
| **Parameter** | n (%) / median (IQR) |
| **Age (years)** | 70 (61 to 77) |
| **Gender** | |
| female | 333 (40.1) |
| male | 497 (59.9) |
| **T stage** | |
| T1 | 131 (15.8) |
| T2 | 264 (31.8) |
| T3 | 364 (43.9) |
| T4 | 70 (8.4) |
| **N stage** | |
| N0 | 440 (53.0) |
| N1 | 291 (35.1) |
| N2 | 99 (11.9) |
| **UICC stage** | |
| UICC I | 281 (33.9) |
| UICC II | 141 (17.0) |
| UICC III | 408 (49.1) |
| **Grading** | |
| G1–2 | 587 (70.7) |
| G3–4 | 243 (29.3) |
| **Lymphovascular invasion** | |
| L0 | 455 (54.8) |
| L1 | 375 (45.2) |
| **Vascular invasion** | |
| V0 | 749 (90.2) |
| V1 | 81 (9.8) |
| **R status** | |
| R0 | 811 (97.7) |
| R1 | 15 (1.8) |
| R2 | 4 (0.5) |
| **Adjuvant therapy** | |
| Surgery Alone | 646 (77.8) |
| Surgery + AC | 184 (22.2) |

**Supplementary Table S2: Chemotherapy regimens used in AC.**

| **Chemotherapy Regimens** | n = 184 |
|---|---|
| | n (%) |
| Capecitabine based | 30 (16.3) |
| Gemcitabine based | 71 (38.6) |
| FOLFIRINOX | 26 (14.1) |
| FOLFOX | 19 (10.3) |
| Not reported | 38 (20.7) |

## Supplementary Figures

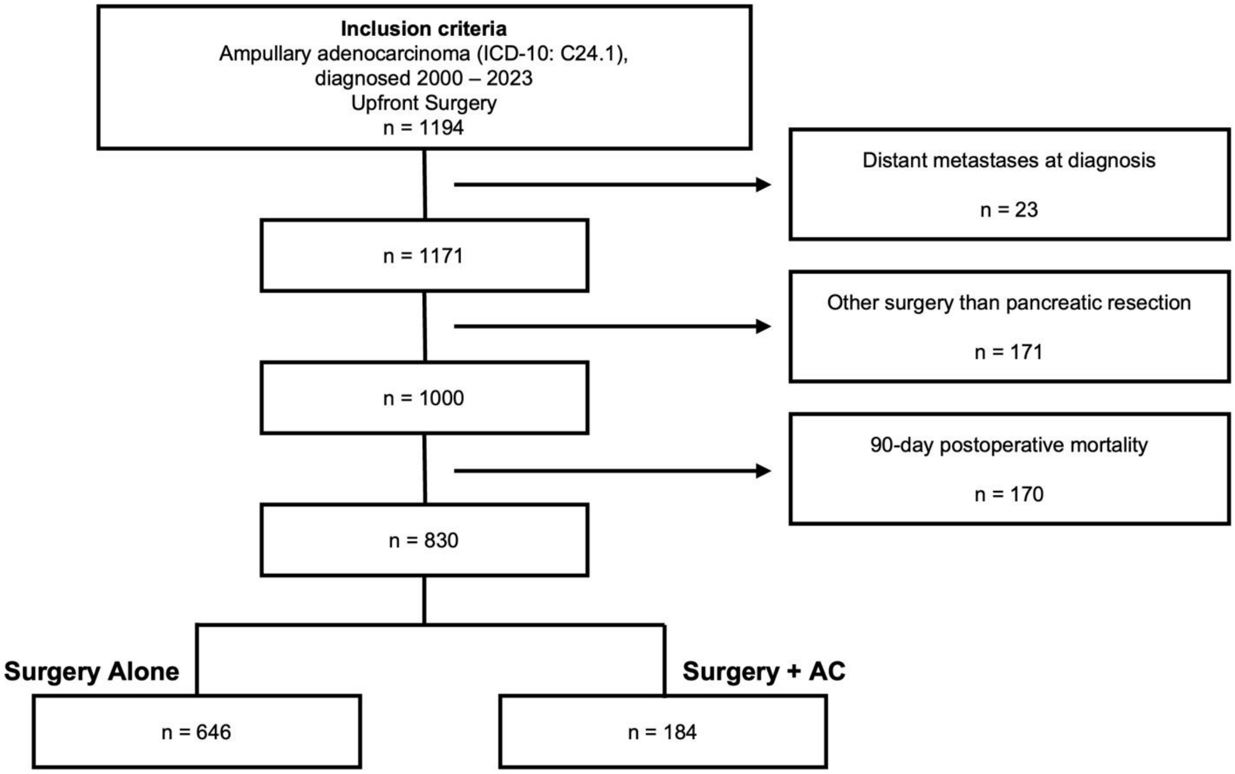


**Supplementary Figure S1: Consort diagram.**

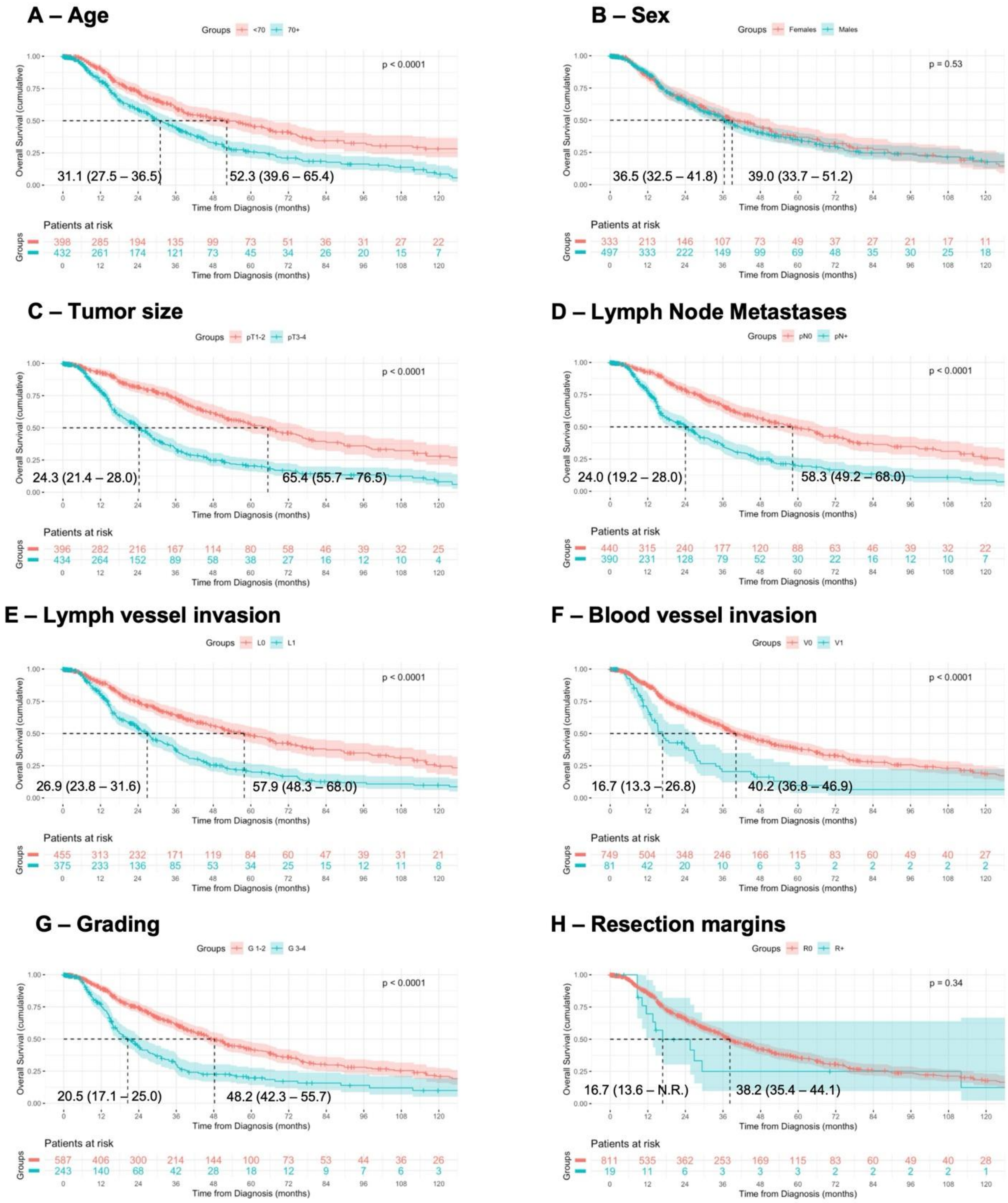


**Supplementary Figure S2: Univariable analysis of prognostic factors for OS.** Association of prognostic factors with OS, using Kaplan-Meier Method with Log-Rank Test, displaying median OS with 95% Confidence interval.

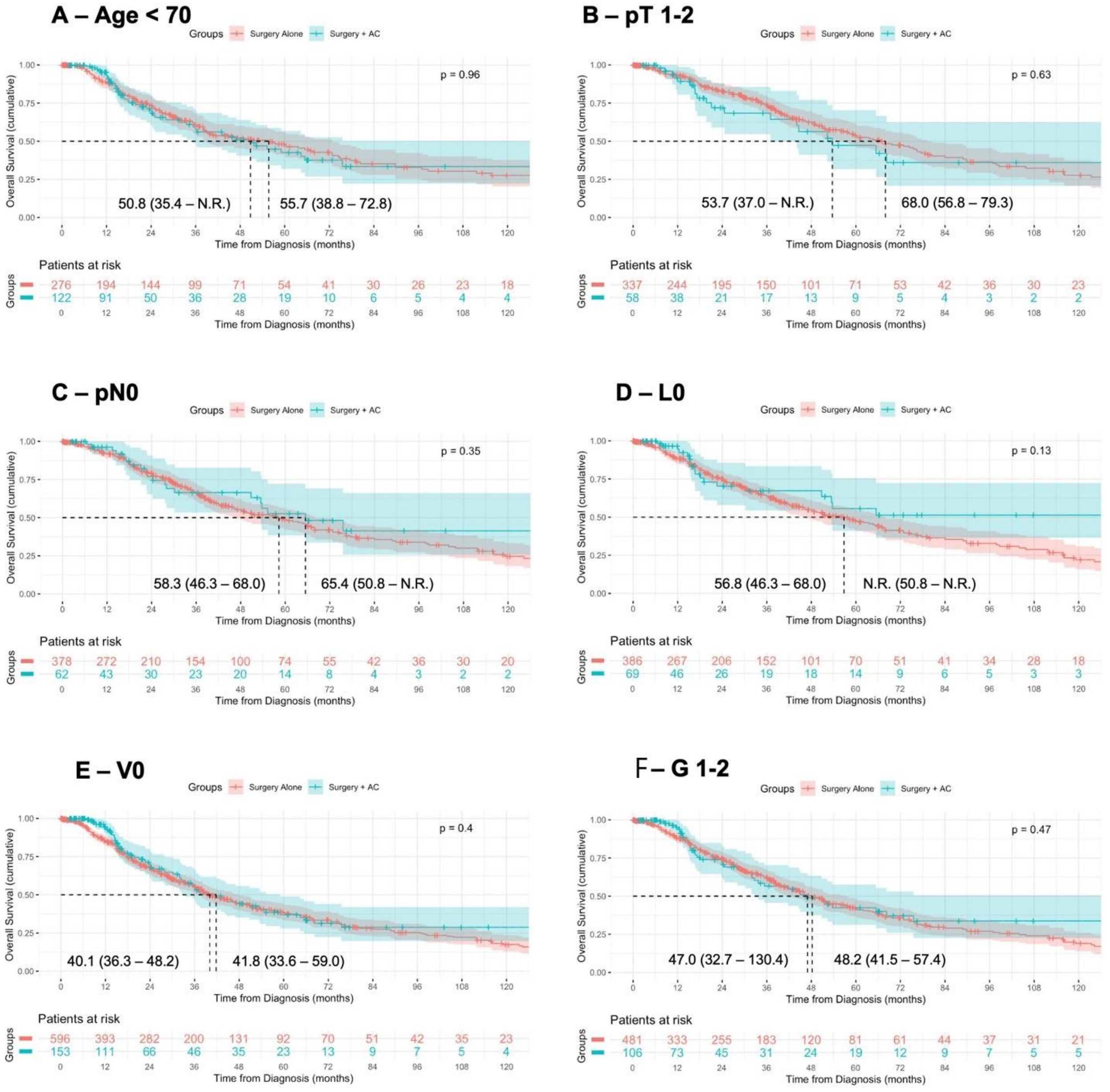


**Supplementary Figure S3: Association of AC with OS in patients with positive prognostic factors.** Comparison of OS from diagnosis in patients with positive prognostic factors receiving Surgery alone or Surgery + AC, using Kaplan-Meier Method with Log-rank test. Displayed is the median OS with 95% Confidence interval.

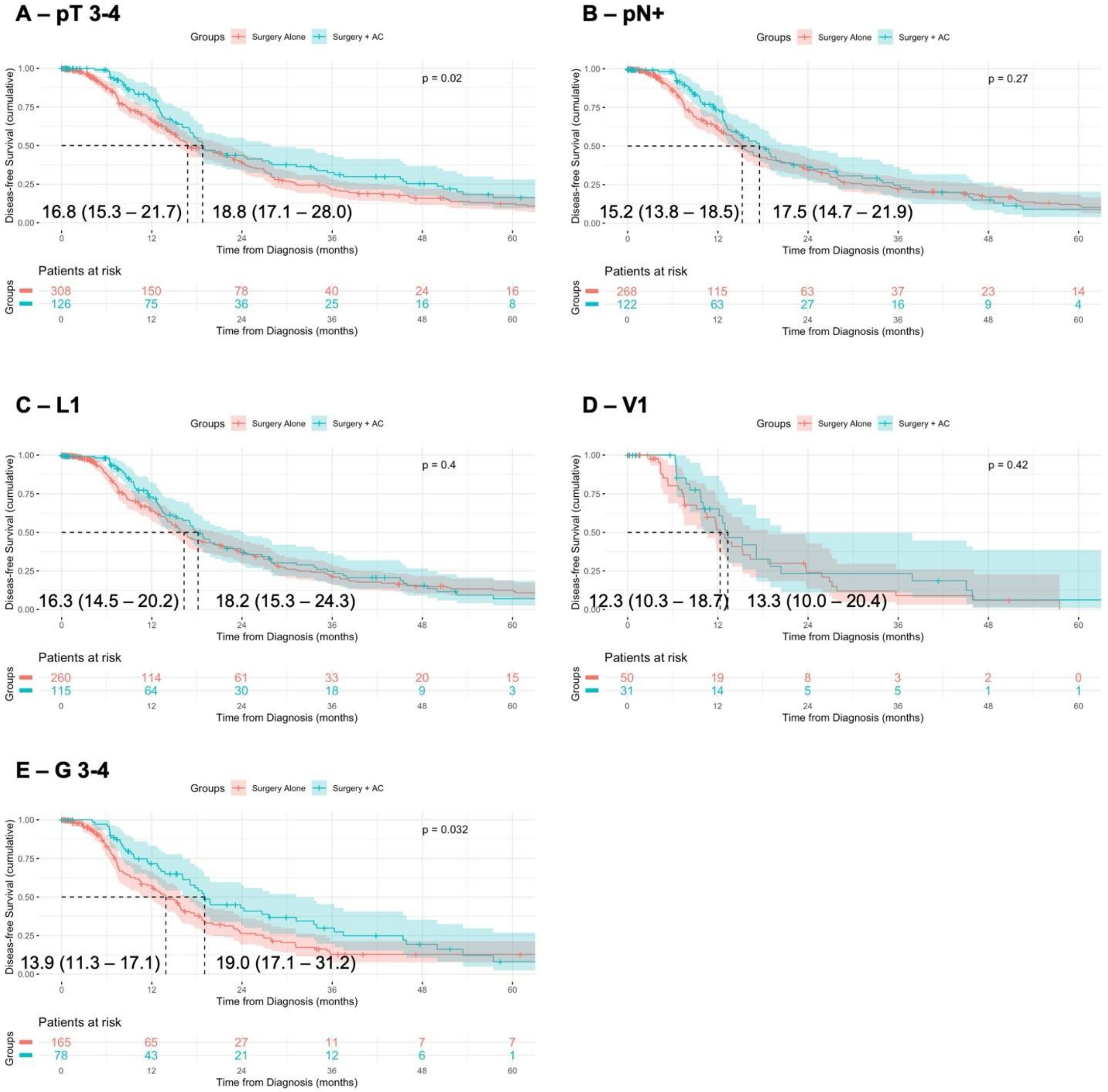


**Supplementary Figure S4: Association of AC with DFS in patients with negative prognostic factors.** Comparison of DFS from diagnosis in patients with negative prognostic factors for DFS receiving Surgery alone or Surgery + AC, using Kaplan-Meier Method with Log-rank test. Displayed is the median OS with 95% Confidence interval.
